# A new cervical nerve root avulsion model using a posterior extra-vertebral approach in rats

**DOI:** 10.1186/1749-7221-8-8

**Published:** 2013-09-11

**Authors:** Takashi Noguchi, Souichi Ohta, Ryosuke Kakinoki, Yukitoshi Kaizawa, Shuichi Matsuda

**Affiliations:** 1 Department of Orthopaedic Surgery, Graduate School of Medicine, Kyoto University, 54 Shogoinkawahara-cho, Sakyo-ku, Kyoto City, Kyoto 606-8507, Japan

**Keywords:** Animal models, Brachial plexus neuropathies, Motor neurons, Nerve root avulsion, Rats

## Abstract

**Background:**

The nerve root avulsion injury causes decrease of motor neurons in the spinal ventral horn. To investigate the motoneuron death after avulsion injury in rats, the intradural root avulsion procedure is usually used, although it is technically demanding and associated with a risk of unexpected spinal cord damage. We have developed a new cervical nerve root avulsion procedure in rats and investigated the validity of our procedure.

**Methods:**

Our procedure is using a posterior approach and pulling the C6 nerve root outside the vertebral foramen without intradural procedures. The lateral third of the lateral mass is needed to be resected before pulling the nerve root. The accomplishment of our procedure is judged by confirmation of the bifurcated stump of the avulsed nerve root and the leakage of the spinal fluid from vertebral foramen. At first, four Sprague–Dawley (SD) rats were used for the examination of C6 motor neuron distribution in the normal spinal cord. Then, 40 SD rats were divided into following four groups and the survival rate of motor neuron was examined. (A) an intradural avulsion group, (B) an intradural rhizotomy group, (C) our extravertebral avulsion group, and (D) an extravertebral rupture group. Another 26 SD rats were used for the examination of histomorphorogic changes in the spinal cord after our extra-vertebral avulsion procedure.

**Results:**

At 28 days after injury, the percentage of surviving motor neurons in groups A (39.0 ± 2.1%) and C (47.5 ± 7.1%) were significantly lower than those in groups B (77.1 ± 12.3%) and D (98.9 ± 9.9%). Compared with other groups, our procedure was easier and associated with less unexpected spinal cord damage. Although the length of the distal stump of the extravertebrally avulsed ventral rootlets was varied between 1.5 and 3.2 mm, this difference did not affect motoneuron death. The extravertebral avulsion injury showed intraspinal bleeding along the motoneuron axons, glial reaction and macrophage infiltration in the lesioned side of the ventral horn.

**Conclusions:**

Our extravertebral avulsion procedure is simple and reproducible. It would become a useful tool for the study of cervical nerve root avulsion injury.

## Background

Severe traction injury to the brachial plexus, may often be followed by the avulsion of one or several nerve roots in the spinal cord. In total brachial plexus nerve root avulsion injury, the function of the affected upper extremity is almost lost completely and permanently. As root avulsion is a longitudinal spinal cord injury, it differs from other peripheral nerve injuries and is considered beyond surgical repair [1]. The current surgical strategy after root avulsion injury is palliative and may include nerve transfer, muscle transfer, and tendon transfer or arthrodesis, resulting in limited and insufficient functional recovery in the injured upper limb [2-4].

Over the past two decades, neurotization of the avulsed nerve root has been reported to be possible in some experimental models by reimplantation of the avulsed nerve root into the spinal cord [5,6]. Subsequently, Carstedt et al. were the first to report reimplantation surgery for avulsed roots in clinical cases [7]. Although this surgical procedure still has a number of unsolved shortcomings, limited functional recovery of the shoulder and elbow joints was seen in some cases. They suggested that one of the important factors for obtaining good functional outcomes in this repair was the survival of motor neurons within the pertinent spinal cord segment [8].

It has been reported that the number of motor neurons in the ventral horn of the spinal cord decreases rapidly after nerve root avulsion injury within a month [9-11]. Loss of neurotrophic support after transection of the peripheral nerve may not only induce apoptosis but also necrotic cell death caused by interference with its vascular supply [12]. Suppression of motor neuron death could be a key to improving the outcome of reimplantation surgery of avulsed nerve roots.

A few rat nerve root avulsion models have been used to investigate the neuroprotective effects of a variety of substances [13-15]. The rat models are mainly categorized into two types: intradural and extra-vertebral procedures. The intradural procedure includes avulsion of the ventral rootlets of the spinal cord exposed by the durotomy following the laminectomy [16]. This procedures is followed by severe intradural and extradural adhesion around the durotomy, even though the dura is closed meticulously.

In extra-vertebral procedures, the nerve roots are pulled directly or indirectly from outside the vertebral column. Several previous authors reported different procedures to produce nerve root avulsion in animal studies. It is difficult for us to predict which nerve roots will be avulsed using the direct traction of the upper arm using a weight-drop device [17], although this model simulates real traction injury of the brachial plexus. Cervical nerve root avulsion via extra-vertebral space using an anterior approach [14] is technically difficult because the subclavian and cervical vessels are located just in front of the brachial plexus.

In the present study, we have developed a less invasive procedure for cervical nerve root avulsion using a posterior extra-vertebral approach in rats and investigated the validity of this new procedure by histological examinations and examining the number of motor neurons remaining in the ventral horn of the spinal cord 28 days after the procedure along with previous reports [9-11].

## Materials and methods

### Animals

Nine-week-old male adult Sprague–Dawley rats (Nippon SLC, Hamamatsu, Japan) weighing about 300 g (280 g to 320 g) were used in this study. All animals were housed in a room with a 14 h light/10 h dark cycle and free access to food and water. Before surgery, rats were anesthetized with sodium pentobarbital intraperitoneally. All animal procedures were approved by the Animal Research Committee, Graduate School of Medicine, Kyoto University (Med Kyo 13237), and performed according to the Guidelines of the Animal Research Committee.

### Distribution of C6 motor neurons

Four rats were used to determine the distribution of C6 motor neurons. The operation was performed on rats placed in a prone position. After detachment of the paraspinal muscles from the C4–Th1 left laminae and anterior articular process (referred to hereafter as “lateral mass” in human anatomy) through a posterior approach, the lateral side of the C5 and C6 lateral mass were exposed. The transverse process and lateral third of the C5 and C6 lateral mass were removed gently using a surgical bur and the C6 nerve root was exposed. Three microliters of 1% hydroxystilbamidine (Invitrogen, Carlsbad, CA, USA), which is the same as Fluoro-Gold solution, was slowly injected into the C6 spinal nerve root using a Hamilton syringe. Two days after the operation, rats were deeply anesthetized and transcardially perfused with 200 ml of phosphate-buffered saline (PBS, pH 7.4) followed by 350 ml of 4% paraformaldehyde (PFA) in 0.1 M phosphate buffer (PB, pH 7.4). A 5-mm-long cervical spinal cord segment was removed from each rat and postfixed overnight in perfusion fixative (4°C) and cryoprotected for 48 h in 20% sucrose in PB (4°C). Two small transverse incisions were made at the lower edge of the C5 dorsal rootlets and at the upper edge of the C7 dorsal rootlets in the contralesional side of the spinal cord. Then, 40-μm-thick serial transverse and coronal sections were obtained from the cervical spinal segments using a cryostat. The distribution of motor neurons in the ventral horn in each section was examined histomorphologically using a fluorescence microscope.

### Surgery

To compare our avulsion model with other brachial plexus injury models, 40 rats were randomly assigned to the following four groups: an intradural avulsion group (group A, n = 10), an intradural rhizotomy group (group B, n = 10), an extra-vertebral avulsion group (group C, n = 10), and an extra-vertebral root rupture group (group D, n = 10). All surgeries were performed with the rat in a prone position under a surgical microscope.

### Intradural avulsion and rhizotomy procedure (group A, B)

A midline skin incision was made in the posterior side of the neck. Left paravertebral muscles of the cervical spine were unilaterally dissected from spinous processes and laminae. Left laminae and the medial part of the lateral mass at C4–C6 were removed with a surgical bur. The dura was opened longitudinally and the left C6 dorsal rootlets were transected using microscissors 0.5 mm distal to the dorsal root entry zone. Then, the left C6 ventral rootlets were pulled from the spinal cord by a traction force applied by a fine microhook (group A) or cut with microscissors as close to the spinal cord surface as possible (group B). All intrathecal distal stumps of the ventral and dorsal rootlets were then removed completely to prevent spontaneous attachment of the residual nerve rootlets to the spinal cord. The dura matter was closed with 10–0 monofilament nylon. The overlying paraspinous muscles and skin were subsequently sutured in layers.

### Our extra-vertebral avulsion procedure (group C)

**Figure 1:**
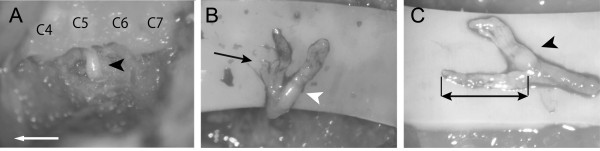
**Intraoperative photographs of the extra-vertebral nerve root avulsion procedure. A**: The left C6 nerve root exposed after resection of lateral third of the lateral mass. The black arrowhead indicates the left C6 nerve root. The white arrow indicates the rostral side. **B**: The distal stump of an avulsed C6 nerve root. The white arrowhead indicates the dorsal rootlets and the black arrow indicates the ventral rootlets. **C**: Resected avulsed spinal nerve root. The black arrow head indicates a dorsal root ganglion. The black double-headed arrow indicates the length of the avulsed ventral rootlets.

A midline skin incision was made in the posterior side of the neck. Left paravertebral muscles of the cervical spine were unilaterally dissected from spinous processes, laminae and lateral mass from the C4–Th1, and the lateral side of the C5 and C6 lateral mass were exposed. The transverse process and lateral third of the C5 and C6 lateral mass were removed gently using a surgical bur. Then, the C6 nerve root was exposed, carefully separated from surrounding ligament-like tissues in the foramen and pulled gently using Adson forceps without teeth (Figure 1A). The accomplishment of our nerve root avulsion procedure is judged by confirmation of the bifurcated stump of the avulsed nerve root and leakage of the spinal fluid from the vertebral foramen (Figure 1B). The leaked spinal fluid often included blood from lacerated vessels accompanying the nerve roots. To stop the leakage of spinal fluid and bleeding, tamponade of the vertebral foramen for some minutes was usually sufficient.

### Extra-vertebral nerve rupture procedures (group D)

After detachment of the paraspinal muscles from the C4–Th1 left laminae and lateral mass, intermuscular dissection was performed beside the lateral side of the C5 and C6 lateral mass and the C6 spinal root was exposed. The C6 nerve root was pulled with the toothless Adson forceps just proximal to the union of C5 and C6 nerve roots without partial resection of the lateral mass or adequate detachment of the nerve root from surrounding tissue as group C. The nerve root was ruptured at the pinched part in the extra-vertebral space. The distal stump was cut away to prevent spontaneous regeneration.

### Length of the distal stump of the avulsed ventral rootlets

Our procedure was unable to confirm directly where the ventral rootlets were ruptured and resulted in several lengths of the distal stump of the avulsed ventral rootlets. Another examination was necessary to evaluate whether the length affected motor neuron loss. Six more rats were used in addition to the extra-vertebral avulsion rats (group C) to evaluate the relationship between the length of the distal stump of the avulsed ventral rootlets and the number of surviving motor neurons (total 16 rats). The ventral C6 nerve root was avulsed using the extra-vertebral avulsion procedure and the length of the avulsed ventral rootlet stump was measured in each rat. In most cases, three stumps were found in each avulsed ventral root. The length of the longest avulsed stump was measured from the spinal root bifurcation of the ventral and dorsal rootlets to the distal end of the stump (Figure 1C). The coefficient of correlation between motor neuron survival ratio at 28 days after the surgery and the length of the avulsed ventral rootlet was calculated.

### Tissue preparation

Rats were deeply anesthetized and transcardially perfused with 200 ml of PBS followed by 350 ml of 4% PFA in 0.1 M PB. The cervical spinal cord segments were removed and postfixed overnight in perfusion fixative (4°C) and cryoprotected for 48 h in 20% sucrose in PB (4°C). Then, C6 spinal segments were cut in a cryostat into 40-μm-thick serial transverse sections.

### Histomorphology of the spinal cord at 12 h after avulsion injury

Eight rats were used to investigate histological changes in the spinal cord at 12 h after the extra-vertebral nerve root avulsion (group C). After fixation with PFA, a transverse section at the C6 spinal cord segment was examined. Sections were then stained with hematoxylin and eosin.

### Motor neuron counting

For motor neuron counting, all sections were stained with 0.5% cresyl violet (Nissl stain). Four weeks after surgery, the number of surviving motor neurons on the lesioned side of the spinal cord was counted in all sections and compared with the number on the unlesioned side. Large multipolar cells with abundant cytoplasm larger than 30 μm in the ventral horn were counted as viable motor neurons [18]. To avoid double counting, the number of the surviving motor neurons was corrected with Abercrombie’s formula [19]. An absence of cell loss would be expressed as 100% of the contralateral control value. Cell counting for all serial sections was performed using a microscope at a final magnification of × 100 by two observers who were blinded to which groups the samples were from. The interindividual correlation was acceptable to analysis.

### Immunohistochemistry

Three primary antibodies were used for immunohistochemistry. The antibodies used were rabbit polyclonal anti-Iba1 (1:2000; Wako, Osaka, Japan), mouse monoclonal anti-ED-1 (1:100; Chemicon, Temecula, CA, USA), and rabbit polyclonal anti-GFAP (1:1000; Chemicon) to identify microglia, macrophages and astrocytes, respectively. Twelve rats were used for immunohistochemistry to detect the glial reaction and immune reaction to our avulsion injury in ventral horn. Three rats were sham-operated where the C5 and C6 lateral masses were partially resected without pulling the nerve root and killed at 14 days after the operation. We used other rats to performed our extra-vertebral avulsion procedure and sacrificed the rats at 14 (n = 4) and 28 days (n = 5) after the operation. Forty-μm-thick transverse frozen sections of the C6 spinal segment were cut as described earlier. Every 12 sections were used for immunohistochemical quantitative analysis of five sections. The staining protocol was performed according to the manufacturer’s protocol (Vector ABC Elite kit; Vector, Burlingame, CA, USA). The sections were pretreated with 0.3% H_2_O_2_ in PBS and preincubated with 2% normal goat serum in 0.2% Triton X-100 in PBS at room temperature. Then, the sections were incubated overnight with the primary antibodies at 4°C. Secondary biotinylated anti-rabbit or anti-mouse antibodies (1:200; Vector) were applied, followed by avidin-biotin-peroxidase complex reagent at room temperature. The immunoreaction was visualized by diaminobenzidine solution and counterstained with hematoxylin. For quantitative measurements, three representative images in the bilateral side of the ventral horn of the C6 spinal segment were captured with a digital camera at a final magnification of × 200. Quantification was performed in a double-blinded fashion with the enhanced contrast and density slicing feature of the Image J software (version 1.33u, NIH, Bethesda, MD, USA). The data were expressed as the number of cells per sections. The double counting of cells was prevented by the distance between sections being preset to 480 μm.

### Statistical analyses

All data were expressed as mean ± SD. The coefficient of correlation between the motor neuron survival ratio at 28 days after the extravertebral avulsion and the length of the avulsed ventral rootlet was calculated by Pearson correlation analysis. The data from motor neuron counting and immunohistochemical findings were compared using one-way analysis of variance followed by a Tukey-Kramer multiple comparisons test (JMP 8, SAS Institute, Cary, NC, USA). Statistical significance was set at P < 0.05.

## Results

### Distribution of C6 motor neurons

**Figure 2:**
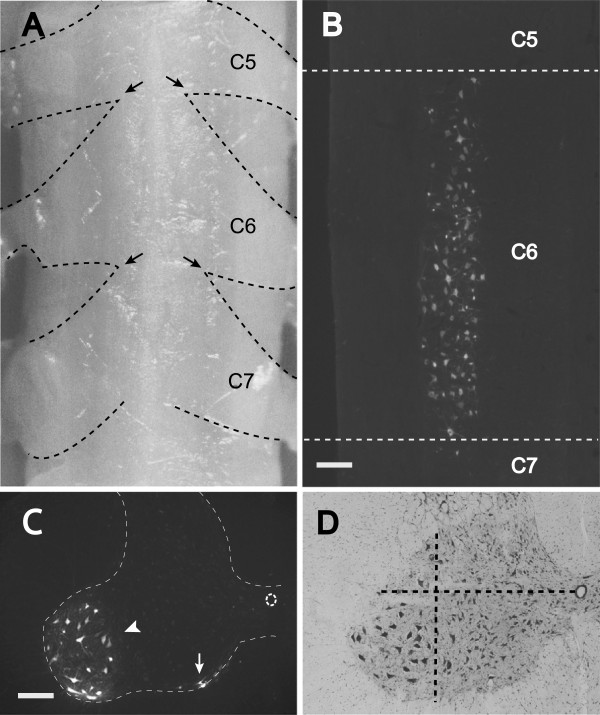
**The distribution of C6 motor neurons. A**: The dorsal view of the cervical spinal cord. The C6 spinal segment was defined as the area between the lower edge of the C5 dorsal rootlets and the upper edge of the C7 dorsal rootlets. Black arrows indicate the border. The dashed lines indicate the contour of the dorsal rootlets. **B**: In the coronal section of the ventral horn of the spinal cord, all hydroxystilbamidine-labeled motor neurons were distributed within the defined C6 segment. **C**: A transverse section of the C6 spinal segment. All hydroxystilbamidine-labeled motor neurons were distributed in the ventral horn. The white arrowhead indicates the motor neurons within the ventrolateral area and the white arrow indicates those within the ventromedial area. The dashed line indicates the border between the white and the gray matter. **D**: The area for motor neuron counting was defined as the area within the gray matter that is enclosed by the two dashed lines (Bar: 200 μm.).

The C6 spinal segment was defined as the area between the lower edge of the C5 dorsal rootlets and the upper edge of the C7 dorsal rootlets (Figure 2A), as previously reported [9]. Motor neurons were retrogradely labeled with hydroxystilbamidine. In the coronal section of the ventral horn of the spinal cord, all labeled motor neurons were distributed within the defined C6 segment (Figure 2B). In the transverse section, most of the labeled motor neurons were found within the ventrolateral area in the gray matter (Figure 2C). Therefore, the area for motor neuron counting was defined as the area within the gray matter that was enclosed by the two lines shown in Figure 2D. One line was a transverse line through the center of the central tube, and the other was a dorsoventral line through the medial-most point of the lateral funiculus. Motor neurons that had more than half of their cell body within the defined area were counted. In cases where cell bodies crossed two of the side limits, motor neurons were counted even if no more than half of the cell body was within the defined area.

### Morphology of the C6 spinal cord segment and the dural sleeve

**Figure 3:**
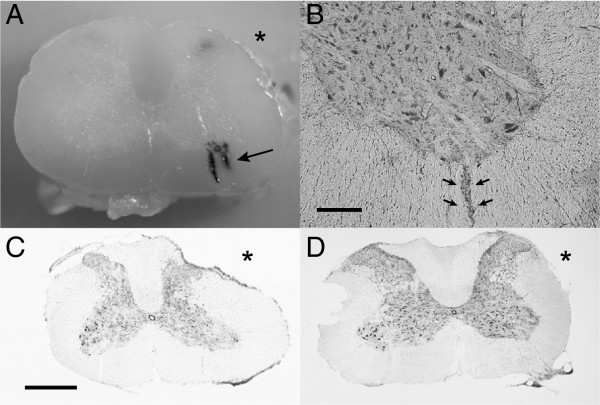
**Histomorphologic changes of the spinal cord after the surgeries. A**: A transverse plane image of the C6 spinal segment at 12 h after extra-vertebral nerve root avulsion. The black arrow indicates the intraspinal bleeding along the motor neuron axons in the ventral spinal cord. The asterisk indicates the lesioned side. **B**: A transverse section of the ventral horn of the lesioned side at 12 h after extra-vertebral avulsion with hematoxylin-eosin staining. The black arrows indicate intraspinal bleeding along the axons of the motor neurons. (Bar: 200 μm.) **C**: A transverse section at 28 days after intradural ventral root avulsion (group A). Spinal cord deformity was found on the lesioned side (*). (Bar: 1 mm.) **D**: A transverse section at 28 days after extra-vertebral nerve root avulsion (group C). The spinal cord was almost intact on the lesioned side (*) (Bar: 1 mm.).

At 12 h after extra-vertebral nerve root avulsion, intraspinal bleeding was found in the white matter along the motor neuron axons in six of eight rats (Figure 3A, B). At 28 days after injury, deformity of the C6 spinal cord segment was found on the lesioned side in the intradural avulsion group and the intradural rhizotomy group (groups A and B) (Figure 3C). Wide adhesion between the dura and the spinal cord was also observed. In contrast, in the extra-vertebral avulsion group and the extra-vertebral rupture group (groups C and D), the deformity and adhesion of the spinal cord were slight (Figure 3D). A cut was made in the lateral funiculus to identify the contralateral unlesioned side. In group B, the mean residual length of the proximal ventral rootlet stump was 1.2 mm. In group C, although the proximal ventral rootlet stumps varied in length, all were within the subarachnoid space. In group D, no ventral rootlets were ruptured or elongated in the subarachnoid space, although the C6 nerve root was lacerated at the pinched point outside the vertebra.

### Length of the distal stump of the avulsed ventral rootlets

**Figure 4:**
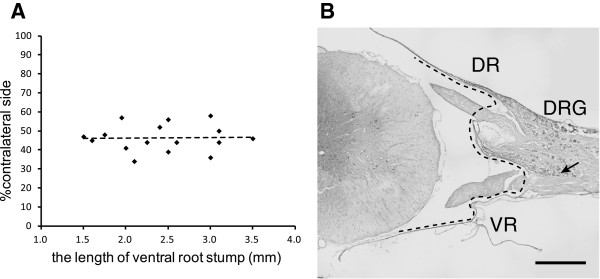
**The rupture point of the ventral rootlets and the motor neuron death. A**: The relationship between the length of the distal stump of the avulsed ventral rootlet and the survival of motor neurons. The coefficient of correlation was 0.03 (P = 0.91). **B**: The relationship between the ventral rootlets and subarachnoid space in the normal C6 spinal cord segment of a 9-week-old male rat. Outer borderline of the subarachnoid space was indicated by a dashed line. The black arrow indicates bifurcation between ventral and dorsal rootlets.

The length of the distal stump of the avulsed ventral rootlets in the extra-vertebral avulsion group (group C) rats ranged from 1.5 to 3.2 mm. The correlation between the length of the avulsed ventral rootlets and the survival of motor neurons is shown in Figure 4A. The coefficient of correlation was 0.03 (P = 0.91). Thus, no significant correlation was found between the length of the distal stump of the avulsed ventral rootlets and the survival of motor neurons. Figure 4B showed the relationship between the ventral rootlets and subarachnoid space in the normal C6 spinal cord segment of 9-week-old male rats. The outer borderline of the subarachnoid space was indicated by a dashed line. The distance between the bifurcation and the subarachnoid space was about 1.0 mm.

### Motor neuron loss

**Figure 5:**
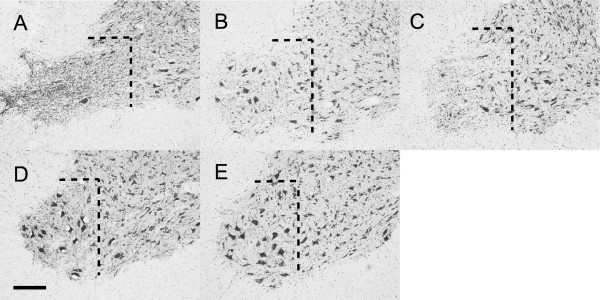
**A representative C6 ventral horn on the lesioned side at 28 days after injury.** Dashed lines indicate landmarks for motor neuron counting. **A**: Intradural avulsion group (group A). **B**: Intradural rhizotomy group (group B). **C**: Extra-vertebral avulsion group (group C). **D**: Extra-vertebral rupture group (group D). **E**: The unlesioned side (control). Compared with E, A and C showed an obvious decrease in the number of motor neurons. B showed a moderate decrease in the number of motor neurons. In D, almost all motor neurons survived (Bar: 200 μm.).

**Figure 6:**
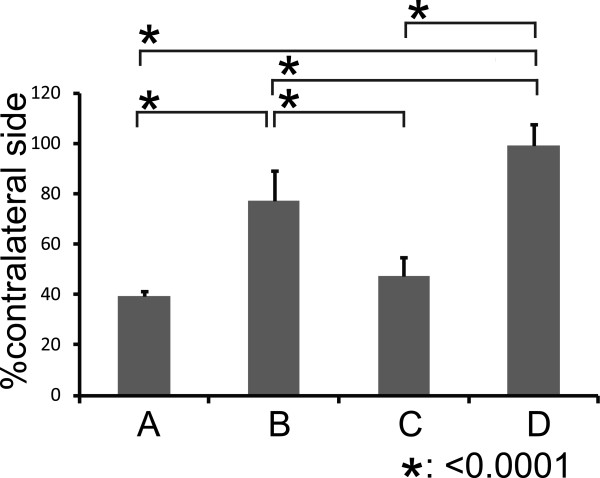
**The percentage of surviving motor neurons in the ventral horn of the C6 spinal segment ((lesioned side/unlesioned side) × 100) at 28 days after injury.** Group A and C rats showed significantly lower numbers of surviving motor neurons than group B and D rats (*P < 0.0001). Group B rats showed a significantly lower number of surviving motor neurons than group D rats (*P < 0.0001). There was no significant difference between group A and C rats (P = 0.30). All data are expressed as mean ± SD.

Within several days after injury, four of the rats in intra-dural avulsion group (group A) had died. Two rats died at one day, one died at two days and another one died at three days after injury. At 28 days after injury, rats in intra-dural avulsion group and extra-vertebral avulsion group (groups A and C) showed markedly decreased survival of motor neurons, whereas almost all motor neurons survived in rats in extra-vertebral rupture group (group D). Intra-dural rhizotomy group rats (group B) showed a slight decrease in the survival of motor neurons (Figure 5). The ratio of surviving motor neurons in the lesioned side compared with the unlesioned side was 39 ± 2.1% in group A, 77.1 ± 12.3% in group B, 47.5 ± 7.1% in group C, and 98.9 ± 9.9% in group D (Figure 6). Rats in groups A and C showed a significantly lower number of surviving motor neurons than those in groups B and D (P < 0.0001). There was no significant difference between groups A and C (P = 0.30).

### Immunohistochemistry

**Figure 7:**
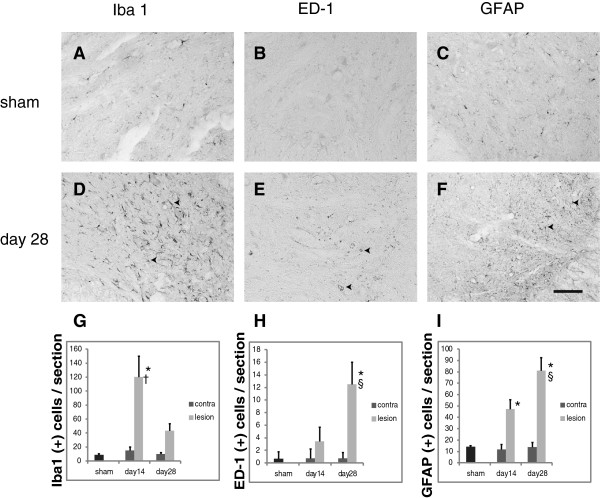
**Immunohistochemical findings.** Immunohistochemistry for Iba1 **(A, D)**, ED-1 **(B, E)**, GFAP **(C, F)** in the ventral horn. Representative sections are shown from sham group **(A, B, C)** and extra-vertebral avulsion group at day 28 **(D, E, F)**. Arrowheads indicate immunopositive cells. (Bar: 100 μm.) **G**, **H**, **I**: Results of quantitative evaluation of immunostaining are summarized in a bar graph (G: Iba1, H:ED-1, I:GFAP). The number of Iba1- and GFAP-positive cells at day 14 and ED-1- and GFAP-positive cells at day 28 significantly increased compared with sham and unlesioned side (*P < 0.001). The number of Iba1-positive cells at day 14 significantly increased compared with at day 28 (^†^P < 0.001). The number of ED-1- and GFAP-positive cells significantly increased at day 28 compared with at day14 (^§^P < 0.001).

In sham-operated rats, the number of Iba1- (microglia), ED-1- (macrophage) and GFAP (astrocyte)-positive cells were minimal in the ventral horn on the lesioned side. In contrast, the root avulsed rats showed remarkably increases of Iba1-, ED-1- and GFAP-positive cells in the ventral horn on the lesioned side at both 14 and 28 days after injury (Figure 7A to 7F). The Iba1-positive cells were significantly greater at day 14 than day 28 (P < 0.001) (Figure 7G). Conversely, the ED-1- and GFAP-positive cells were significantly greater at day 28 than day 14 (P < 0.001) (Figure 7H, I). Iba1-positive cells and ED-1-positive cells were found not only in the ventral horn but also in the white matter around the motor neuron axons.

## Discussion

Cervical nerve root avulsion models in rats have been used in several studies, most of them using an intradural root avulsion procedure [20-22]. In the present study, the number of surviving motor neurons in the intradural root avulsion group was significantly decreased at 28 days after injury. However, the mortality rate of the rats in the intradural root avulsion group was distinctly higher within several days after injury compared with the other groups. Immediately after the intradural root avulsion procedure, bleeding and edema were often observed in the operated spinal cord. The simultaneous rupture of arteries accompanying the ventral rootlets and the direct traction force to the ventral rootlets applied by the microhook used in the procedure might cause severe damage not only to the motor neurons, but also the surrounding tissues in the spinal cord. Therefore, we consider the intradural avulsion procedure to be inappropriate and too invasive for a model of nerve root avulsion injury.

The survival of spinal motor neurons following rhizotomy in adult rats was reported to be mainly dependent on the length of the remaining axons, because neurotrophic factors produced by the non-neuronal cells associated with the remaining axons might prevent the injured motor neurons from dying [23]. Our result after the rhizotomy was consistent with the report. However, in our extra-vertebral avulsion group, there was no correlation between the length of the avulsed ventral rootlet stump and the survival of motor neurons. The shortest length of the distal stump of the avulsed ventral rootlets was 1.5 mm. In the normal spinal cord of 9-week-old male rats, the point 1.5 mm proximal from the union of ventral and dorsal rootlets was within the subarachnoid space. Within the dural sleeve, the ventral rootlets run in contact with the dorsal root ganglia and dura matter. In contrast, within the subarachnoid space, the ventral rootlets are separated from surrounding tissues and tend to be more fragile than those within the dural sleeve. Therefore, a traction force to the nerve root would cause rupture of the ventral rootlets within the subarachnoid space where the rootlets are the most fragile. The confirmation of the bifurcated stump of the avulsed nerve root is a simple and effective way to assess the rupture of the ventral rootlets in our extra-vertebral avulsion procedure.

After nerve root avulsion injury, intraspinal bleeding was found in the white matter along the motor neuron axons. During the avulsion procedure, the maximum tensile strength of the ventral rootlets would be loaded to the motor neurons just before rupture of the rootlets. Although the ventral rootlets were disconnected within the subarachnoid space in rats in both the intradural rhizotomy group and the extra-vertebral avulsion group, the extra-vertebral avulsion group showed a significant decrease in motor neurons compared with the intradural rhizotomy group. The traction force to the nerve rootlets would cause severe injury to the motor neurons and affect the motor neuron survival rate. Some authors demonstrated the neuroprotective effects of growth factors on motor neurons using nerve root avulsion models and suggested that these factors offered a possible new treatment for neurodegenerative diseases, such as amyotrophic lateral sclerosis [24,25]. Given the traction injury to the motor neurons, nerve root avulsion models can be used for the study of neurodegenerative diseases but with some limitations.

Our extra-vertebral nerve root avulsion procedure has two advantages. First, this procedure causes no unexpected mechanical damage to the spinal cord. In the intradural procedures, it is important to preserve the dura matter so that it can be repaired after the ventral root avulsion. Therefore, because of the very narrow working space, the spinal cord was often somewhat damaged, even if the operation was performed under a microscope. Moreover, the intradural procedure causes spinal cord deformity and extensive adhesion between the dura matter and the spinal cord [13,26], even if the dura matter is meticulously repaired. Unexpected factors, such as spinal cord damage or adhesion around the spinal cord, should be eliminated as much as possible to study the effects of various methods or substances on motor neurons or glial reactions after nerve root avulsion injury. Our extra-vertebral avulsion procedure, which preserves the lamina and dura, would be ideal for such studies.

Second, our procedure does not involve complex or difficult techniques. The targeted nerve root was easily found using the second thoracic vertebra with the longest spinal process as a landmark. The most important point in our procedure is adequate removal of the ligament-like tissues similar to transverse radicular ligament around the nerve root in the vertebral foramen [27]. This was easily achieved by resection of the lateral third of the lateral mass. An anterior extra-vertebral approach was used in some other studies [9,25,28]. We attempted this anterior approach and found a serious problem. Although the distal part of the cervical nerve root was able to be identified by splitting the pectoralis major muscle through the anterior approach, exploration of the vertebral foramen to remove the ligament-like tissues around the nerve root was very difficult because of the existence of the cervical artery and vein in front of the brachial plexus.

In the present study, we defined the area for motor neuron counting using simple landmarks. To our knowledge, no other reports show clear borderlines of the area for motor neuron counting in transverse sections. According to Rexed’s report [29], spinal motor neurons distribute in both the wider ventrolateral area and the smaller ventromedial area of the anterior horn. Motor neurons in the ventromedial area and those in the ventrolateral area innervate the muscles in the trunk and the extremities, respectively. Although our defined motor neuron counting area excluded the small ventromedial area, a decrease in the number of motor neurons after the injury was shown as previously reported in other root avulsion models. In the present study, the area for motor neuron counting for the C6 spinal cord segment is defined. Although the axial pattern of gray matter in other cervical spinal cord segments is almost similar to that of the C6 segment, those in the thoracic and lumbar spinal cord segments are different. Therefore, the definition of the area for motor neuron counting given in the present study cannot be used for other spinal segments. Moreover, the severe deformity of the spinal cord following other avulsion procedures, such as those after intradural avulsion, might hinder motor neuron counting.

In our nerve root avulsion procedure, the exact traction force of the nerve roots was not established as it was in previous reports that described the manner of pulling the nerve rootlets in the subarachnoid space as “slowly, moderate, or mild” [30-32]. However, only removal of the lateral third of the lateral mass and ligament-like tissues around the nerve root enabled us to perform the avulsion easily by gentle extra-vertebral pulling of the nerve roots, which was not very strong and avoided them being ruptured outside the vertebral foramen. Furthermore, in our procedure, so long as the bifurcated stump of the avulsed nerve root and the leakage of the spinal fluid from vertebral foramen were confirmed, the procedure is considered to be successful. Therefore, determination of the detailed traction force was unnecessary in our procedure.

## Conclusions

A new rat model for cervical nerve root avulsion with a posterior extra-vertebral approach is reported herein. Compared with other nerve root avulsion models, this procedure is easier and associated with less unexpected spinal cord damage. The accomplishment of this procedure is only judged by confirmation of the bifurcated stump of the avulsed nerve root and the leakage of the spinal fluid from vertebral foramen. This procedure may be useful for more precise study of the effects of neuroprotective substrates or procedures on cervical nerve root avulsion injury in rats.

## Abbreviations

PFA: Paraformaldehyde; PBS: Phosphate buffered saline; PB: Phosphate buffer; DRG: Dorsal root ganglion; VR: Ventral root; DR: Dorsal root; SD: Standard deviation.

## Competing interests

The authors declare that they have no competing interests.

## Authors’ contribution

Conceived and designed the experiments: SO and SM. Performed the experiments: TN and SO. Analyzed the data: SO. Performed the cell counting: YK and RK. Wrote the paper: TN and SO. All authors read and approved the final manuscript.
